# Preoperative assessment of intratumor heterogeneity using intravoxel incoherent motion MRI for survival prediction in high-grade gliomas: a feasibility study

**DOI:** 10.1186/s12880-026-02499-6

**Published:** 2026-06-12

**Authors:** Xingrui Wang, Yuanzheng Wang, Xiaoqing Wang, Fang Liu, Shiteng Suo, Yang Song, Yan Zhou, Mengqiu Cao

**Affiliations:** 1 Department of Radiology, Renji Hospital, Shanghai Jiao Tong University School of Medicine, Shanghai, 200127 China; 2 Department of Radiology, Tongren Hospital, Shanghai Jiao Tong University School of Medicine, Shanghai, 200336 China; 3 MR Research Collaboration Team, Siemens Healthineers Ltd, Shanghai, 200126 China; 4 College of Health Science and Technology, Shanghai Jiao Tong University School of Medicine, Shanghai, 200025 China

**Keywords:** Intravoxel incoherent motion, Magnetic resonance imaging, High-grade glioma, Intratumor heterogeneity, Prognosis

## Abstract

**Background:**

Intratumor heterogeneity (ITH) is closely associated with poor prognosis in high-grade gliomas (HGGs). This study aimed to characterize ITH and explore potential imaging markers that predict overall survival (OS) in HGGs using intravoxel incoherent motion magnetic resonance imaging (IVIM MRI)-based spatially explicit analysis.

**Methods:**

Sixty-five HGG patients who underwent surgical resection were analyzed. Preoperative IVIM MRI images were collected and processed to obtain true diffusion coefficient (*D*) and perfusion fraction (*f*) maps. Tumor regions of interest were segmented, and the k-means algorithm was applied to cluster the *D* and *f* image voxels for generating spatial habitats and extracting quantitative image features. Kaplan-Meier analysis and Cox proportional hazards were used to compare variables and patient subgroups.

**Results:**

Three spatial habitats were identified: Habitat 1 (hypo-vascular, hyper-cellular), Habitat 2 (hypo-cellular), and Habitat 3 (hyper-vascular). In the multivariate Cox regression analysis, isocitrate dehydrogenase (IDH) genotype (hazard ratio [HR] = 0.298, *P* = 0.003) and volume percentage (pVol) of Habitat 1 (HR = 6.155, *P* = 0.01) showed prognostic significance, with the model yielding a concordance index of 0.756. A pVol value of Habitat 1 below 47.6% predicted survival benefits in patients with HGG and IDH wild-type gliomas, as well as in those with HGG who underwent subtotal resection (median OS improvement: 11, 11, and 8 months, respectively).

**Conclusions:**

Spatial habitats identified via IVIM MRI may aid in characterizing cellular and vascular heterogeneity in HGGs, with the pVol of hypo-vascular, hyper-cellular habitat potentially serving as an independent predictor of patient survival.

**Supplementary Information:**

The online version contains supplementary material available at 10.1186/s12880-026-02499-6.

## Introduction

Gliomas are the most prevalent primary brain tumors with a poor prognosis in adults [1]. Current treatments offer limited benefits due to intratumor heterogeneity (ITH), especially in high-grade gliomas (HGGs) [2, 3]. ITH is closely correlated with the poor prognosis of HGG, and heterogeneity at the molecular level is one of the key factors contributing to ITH [4–6]. Genetic and microenvironmental factors create ITH habitats containing subpopulations of cellular and vascular components with distinct physiological characteristics [3]. Clinically, the assessment of glioma prognosis primarily depends on pathological and molecular characteristics [1, 7], the accurate determination of which typically requires surgery or biopsy. However, these methods are invasive, time-consuming, and subject to sampling bias introduced by ITH. There remains a need for developing preoperative noninvasive biomarkers to assess ITH and prognosis in HGGs.

Spatially explicit analysis, also known as habitat imaging, is an advanced image-processing framework that leverages various algorithms to analyze multiparametric imaging data, including magnetic resonance imaging (MRI) [4]. This technique enables the identification and characterization of distinct intratumor habitats based on their biological attributes and physiological relevance [4, 8]. Data-driven approaches in habitat analysis have facilitated an integrated assessment of tumor heterogeneity and quantitative characterization of specific subregions [9]. Recent studies have applied this technique to glioma imaging and demonstrated its promising capability in delineating ITH in relation to key biological properties, including perfusion dynamics and cellular density [10, 11].

In the past few years, increasing attention has been directed toward advanced MRI techniques for tumor evaluation, particularly in efforts to improve the characterization of tumor physiology and heterogeneity. These approaches broadly include both contrast-based and non-contrast imaging methods. For example, the concept of fractional tumor burden (FTB), typically derived from contrast-enhanced perfusion imaging, enables voxel-wise stratification of tumor subregions based on vascular characteristics, thereby providing insights into tumor aggressiveness, treatment response, and spatial heterogeneity [12, 13]. In parallel, non-contrast MRI techniques such as arterial spin labeling (ASL) has been developed to quantify cerebral blood flow without the use of exogenous contrast agents, offering a non-invasive means of assessing tumor perfusion [14]. Despite their respective advantages, these techniques also present inherent limitations. FTB relies on contrast-enhanced acquisitions and predefined thresholds, which may introduce variability across studies, whereas ASL is often constrained by a relatively low signal-to-noise ratio and limited spatial resolution [13].

Within the non-contrast MRI framework, intravoxel incoherent motion (IVIM) MRI represents a promising complementary approach, as it enables the simultaneous assessment of diffusion and microvascular perfusion components using a unified acquisition without contrast agents [15]. It provides a unique opportunity to further characterize ITH, particularly in settings requiring longitudinal monitoring or in patients with contraindications to contrast agents [15, 16]. By applying different levels of diffusion weighting (*b* values), IVIM simultaneously evaluates capillary microcirculation and the molecular diffusion of water within tissues by calculating specific quantitative parameters, such as the perfusion fraction (*f*) and the true diffusion coefficient (*D*), respectively [17, 18]. Notably, both perfusion and cellularity play a crucial role in the emergence and progression of ITH and have been shown to be closely associated with the molecular characteristics and biological behavior of HGGs [19–21].

In this study, we hypothesized that tumor regions with distinct vascular and cellular characteristics could be identified using spatially explicit IVIM MRI analysis. Accordingly, we aimed to characterize intratumor vascular and cellular heterogeneity in HGGs and to identify imaging biomarkers that may preoperatively predict overall survival (OS).

## Materials and methods

### Patients

The local institutional review board approved this retrospective study, and the requirement for informed consent was waived due to its retrospective nature. Patients with newly diagnosed glioma who underwent surgical resections in the neurosurgery department in our hospital between January 2018 and January 2020 were screened. The inclusion criteria were: (a) histopathologic-confirmed HGG (WHO grade 3–4) according to the 2021 WHO classification of central nervous system (CNS) tumors, with known isocitrate dehydrogenase (IDH) genotypes confirmed by Sanger sequencing; (b) baseline multiparametric MRI, including IVIM; (c) no prior history of glioma treatments before MRI, including surgery, radiation therapy, chemotherapy, or corticosteroid treatment; (d) standard-of-care treatment for HGG, following MRI, including gross total resection (GTR) or subtotal resection (STR), followed by radiation therapy and/or chemotherapy. Of the 97 initial patients, 32 were excluded: 3 were younger than 18 years of age, 5 had severe image artifacts on IVIM images, and 24 were lost to follow-up. A total of 65 patients were finally included. The OS was calculated from the date of initial diagnosis to death or the last follow-up visit (censored events).

### Image acquisition

All images were obtained using a 3.0T MR scanner (MAGNETOM Prisma, Siemens Healthcare, Erlangen, Germany) with a 20-channel head coil. Standard scanning sequences included unenhanced T1-weighted imaging (T1WI) [repetition time (TR)/time of echo (TE) = 137/2.5 ms, matrix = 320 × 290, slice thickness = 5 mm, field of view (FOV) = 199 mm × 220 mm], T2-weighted imaging (T2WI) [TR/TE = 4500/103 ms, matrix = 384 × 348, slice thickness = 5 mm, FOV = 199 mm × 220 mm], T2-weighted fluid attenuation inversion recovery (FLAIR) [TR/TE = 8000/86 ms, matrix = 320 × 290, slice thickness = 5 mm, FOV = 199 mm × 220 mm], and contrast-enhanced T1WI [TR/ TE = 137/2.5 ms, matrix = 320 × 290, slice thickness = 5 mm, FOV = 199 mm × 220 mm]. A standard dose (0.1 mmol/kg) of gadopentetate dimeglumine (Magnevist; Bayer Healthcare) was administered intravenously at a rate of 4 ml/s.

IVIM MRI was performed using spin-echo echo-planar DWI with multiple *b*-values (*b* = 0, 20, 50, 80, 100, 200, 500, 800, and 1000 s/mm^2^) in three orthogonal directions. The imaging parameters were: TR/TE = 6500/74 ms, matrix = 192 ⋅ 192, FOV = 240 mm ⋅ 240 mm, slice thickness = 4 mm, number of slices = 34. IVIM imaging was performed prior to contrast agent injection. The total acquisition time for the IVIM scanning was 4 min and 9 s.

### Image processing

####  IVIM model

IVIM image processing was performed using the open-source software Medical Imaging Interaction Toolkit (MITK, version 5.2.1, German Cancer Research Center, Heidelberg, Germany; https://www.MITK.org). The diffusion-weighted signal decay was modeled using the standard bi-exponential IVIM equation [17]:

$$\:\frac{{SI}_{b}}{{SI}_{0}}=\left(1-f\right)\times\:exp\left(-bD\right)+f\times\:exp\left(-b{D}^{*}\right)$$

where *SI*_*b*_ and *SI*_*0*_ are the signal intensities at a given b-value and at b = 0 s/mm², respectively; *D* is the true molecular diffusion coefficient, *f* represents the fractional volume of microvascular perfusion within a voxel, and D^*^ is the pseudo-diffusion coefficient reflecting microvascular flow. In this study, D^*^ was included in the fitting procedure but is recognized to be sensitive to low b-value sampling and noise, as commonly reported [17, 22]. Multiple b-values were employed to adequately capture both perfusion-related signal decay (b < 200 s/mm²) and diffusion-related decay (b ≥ 200 s/mm²), ensuring robust estimation of *D* and *f* while acknowledging potential instability in D^*^.

#### Tumor segmentation

All tumor regions of interest (ROIs) were manually delineated by a radiologist (X.R.W., with 8 years of neuroimaging experience) on DWI (b = 0 s/mm²) images, slice-by-slice, using the level tracing segmentation method in 3D Slicer software (version 4.8.1, https://www.slicer.org). DWI was selected as the primary segmentation reference to ensure spatial consistency with IVIM-derived parametric maps, as both originate from the same acquisition space, thereby minimizing potential misregistration errors in voxel-wise analysis. Each ROI covered the solid tumor component, excluding areas of necrosis, cysts, hemorrhage, peritumoral edema, and prominent non-tumor macro-vessels. T1WI, T2WI, FLAIR and contrast-enhanced-T1WI (ce-T1) images were used to confirm the solid components and to exclude non-tumor parenchymal areas. The ROIs delineated by X.R.W. were reviewed for quality control by a senior neuroradiologist (M.Q.C., with 15 years of experience in CNS tumors), and any uncertainties regarding ROI boundaries were resolved by consensus. The quality-controlled ROIs were used for subsequent feature extraction. To assess interobserver reproducibility, another neuroradiologist (Y.Z., with 15 years of experience in CNS tumors) independently delineated tumors in a randomly selected subset of 20 patients. All radiologists were blinded to the patients’ clinical, pathological, or molecular information.

#### Habitat mapping and feature extraction feature extraction

Habitat mapping was performed using FeAture Explorer software (FAE, version 0.5.12, Shanghai Key Laboratory of Magnetic Resonance; https://github.com/salan668/FAE.git) [23]. Voxel-wise clustering was achieved via the k-means algorithm, which assigns each voxel to the nearest cluster centroid. The software was configured in cohort-based mode, enabling aggregation of voxel-level data across the entire patient cohort for unsupervised clustering. Prior to clustering, both *D* and *f* maps were standardized using cohort-wise *Z*-score normalization, based on the mean and standard deviation computed across the full cohort, thereby removing inter-patient scale variations while preserving relative voxel-wise differences. Tumor ROIs were then mapped onto the *D* and *f* images, and voxels within these masks were extracted for subsequent habitat analysis. Each voxel was characterized by two features: *D* and *f* value. To determine the optimal number of clusters, we evaluated k-means solutions with cluster counts ranging from 2 to 5, considering both the sum of squared errors (SSE) and silhouette scores (Supplemental Fig. 1). We chose k = 3, which balances cluster cohesion, separation, and biological interpretability while maintaining model simplicity and reducing the risk of over-parameterization [24].

Therefore, a tripartite segmentation of the voxel collection was established. This segmentation identified three distinct habitats:

1. the hypo-vascular, hyper-cellular habitat (Habitat 1), characterized by low f and low D values;
2.  the hypo-cellular habitat (Habitat 2), characterized by high D values;
3. the hyper-vascular habitat (Habitat 3), characterized by high f values

**Fig. 1:**
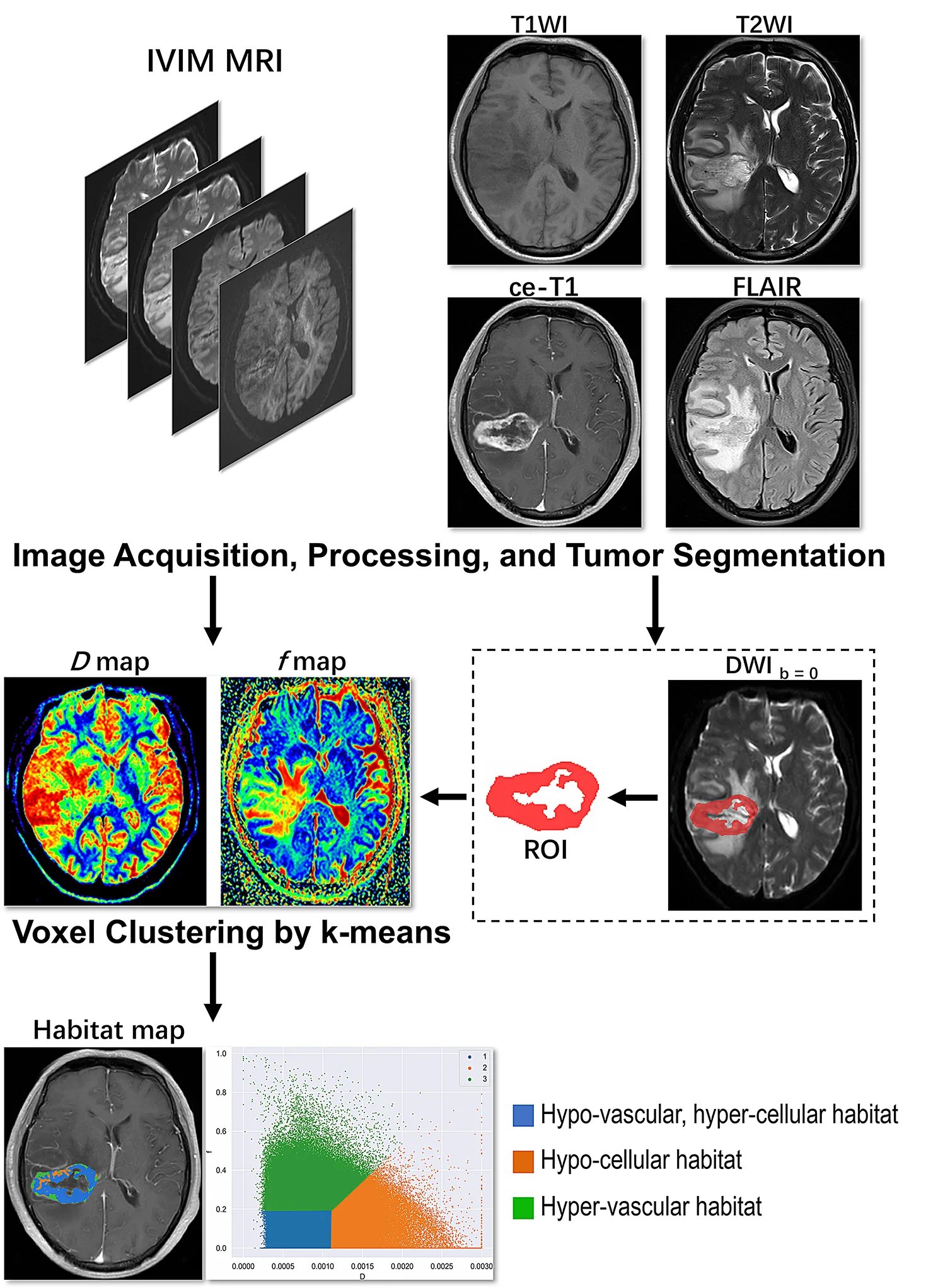
The flowchart of data processing. IVIM, intravoxel incoherent motion; MRI, magnetic resonance imaging; T1WI, T1-weighted imaging; T2WI, T2-weighted imaging; ce-T1, contrast-enhanced T1-weighted imaging; FLAIR, fluid attenuation inversion recovery; *D*, true diffusion coefficient; *f*, perfusion fraction; ROI, region of interest; DWI, diffusion-weighted imaging

The flowchart of data processing is shown in Fig. 1. After clustering the image voxels, the following quantitative features were calculated: the mean *f* value (*f*_mean_) and mean *D* value (*D*_mean_) in each habitat, as well as the volume and volume percentage (pVol) of each habitat within the tumor ROIs. Both *f*_mean_ and *D*_mean_ within each tumor ROI were also extracted.

### Statistical analysis

Statistical analyses were performed using SPSS (version 26.0; IBM, Armonk, NY), MedCalc (version 22.001, Ostend, Belgium), and R software (version 4.3.3, R Foundation). The normality of quantitative variables was assessed using the Kolmogorov-Smirnov test. Inter-observer agreement of tumor segmentation was evaluated using Dice similarity coefficient, with an acceptable threshold defined as ≥ 0.8.

Univariate Cox proportional hazards regression analysis was initially conducted to identify features associated with OS. The optimal cutoff value for each predictor was determined by selecting the threshold that minimized the log-rank *P* value [25]. Variables with *P* < 0.05 in the univariate analysis were entered into the multivariate Cox regression model using a stepwise selection procedure. Multicollinearity among predictors was assessed using the variance inflation factor (VIF). Three multivariable Cox regression models were constructed: a clinical model (Model C), a clinical-ROI model (Model CR), and a clinical-habitat model (Model CH). Model performance was evaluated using Harrell’s concordance index (C-index) and Akaike information criterion (AIC). The 95% confidence intervals (CIs) for the C-index were estimated using percentile bootstrap resampling with 1,000 iterations. Comparative model performance was assessed using paired bootstrap analyses with 1,000 resamples, with statistical significance determined from the empirical distribution of C-index differences.

Based on the cutoff values of significant predictors, patients were stratified into high- and low-risk groups. Kaplan-Meier survival curves and log-rank tests were used to compare OS between the two groups. Hazard ratios (HR) and 95% confidence interval (CI) were calculated for each group. A two-tailed *P* value < 0.05 was considered statistically significant.

## Results

### Patient characteristics

Patient characteristics are summarized in Table 1. Our study cohort comprised 65 patients with HGG, all of whom underwent standard surgical resection. During a mean follow-up period of 17.1 ± 12.6 months (range, 0–36 months), 49 (75.4%) of the 65 patients died, including 9 (18.4%) with IDH mutations and 40 (81.6%) with IDH wild-type. Of these, 17 (34.7%) underwent GTR, and 32 (65.3%) underwent STR.

**Table 1:** Patient characteristics

Characteristic	HGG (*n* = 65)
Age (years)	
Mean ± SD	56.35 ± 14.51
Sex, male/female	41 / 24
WHO grade, n (%)	
3	13 (20.0%)
4	52 (80.0%)
IDH genotype, n (%)	
IDH mutant	19 (29.2%)
IDH wild-type	46 (70.8%)
Resection type, n (%)	
GTR	26 (40.0%)
STR	39 (60.0%)
Follow-up time (months)	
Mean ± SD	17.1 ± 12.6
Median (IQR)	14.0 (6.0–30.0)

HGG, high-grade glioma; IDH, isocitrate dehydrogenase; GTR, gross total resection; STR, subtotal resection; SD, standard deviation; IQR, interquartile range.

### Inter-observer reproducibility and habitat-specific features

Inter-observer agreement for tumor segmentation was excellent, with a mean Dice similarity coefficient of 0.846 ± 0.031. Individual Dice similarity coefficients for the randomly selected subset of 20 patients are provided in Supplementary Table S1.

Quantitative characteristics of the IVIM-derived habitats are summarized in Table 2, demonstrating distinct diffusion-, perfusion-, and volume-related profiles among the three habitats.

**Table 2:** Quantitative Features of IVIM-Derived Habitats and the Entire Tumor ROI

Parameter	Habitat 1	Habitat 2	Habitat 3	Tumor ROI
*D*_mean_ (×10⁻³ mm²/s)	0.812 (0.695–0.906)	1.384 (1.292–1.509)	0.782 (0.682–0.877)	0.916 (0.743–1.086)
*f*_mean_ (%)	10.35 (6.75–14.03)	8.93 (3.65–17.91)	25.96 (24.26–29.26)	14.79 (7.56–22.04)
Volume (cm^3^)	15.53 (9.97–31.71)	7.09 (1.65–14.02)	6.28 (1.79–23.40)	41.06 (25.05–75.07)
pVol (%)	48.2 (23.6–64.3)	13.4 (4.5–32.4)	19.6 (5.2–55.7)	N/A

Data are presented as median (interquartile range). IVIM, intravoxel incoherent motion; ROI, region of interest; *D*, true diffusion coefficient; *f*, perfusion fraction; pVol, volume percentage; N/A, not applicable.

### Habitat features from IVIM MRI predict OS

**Fig. 2:**
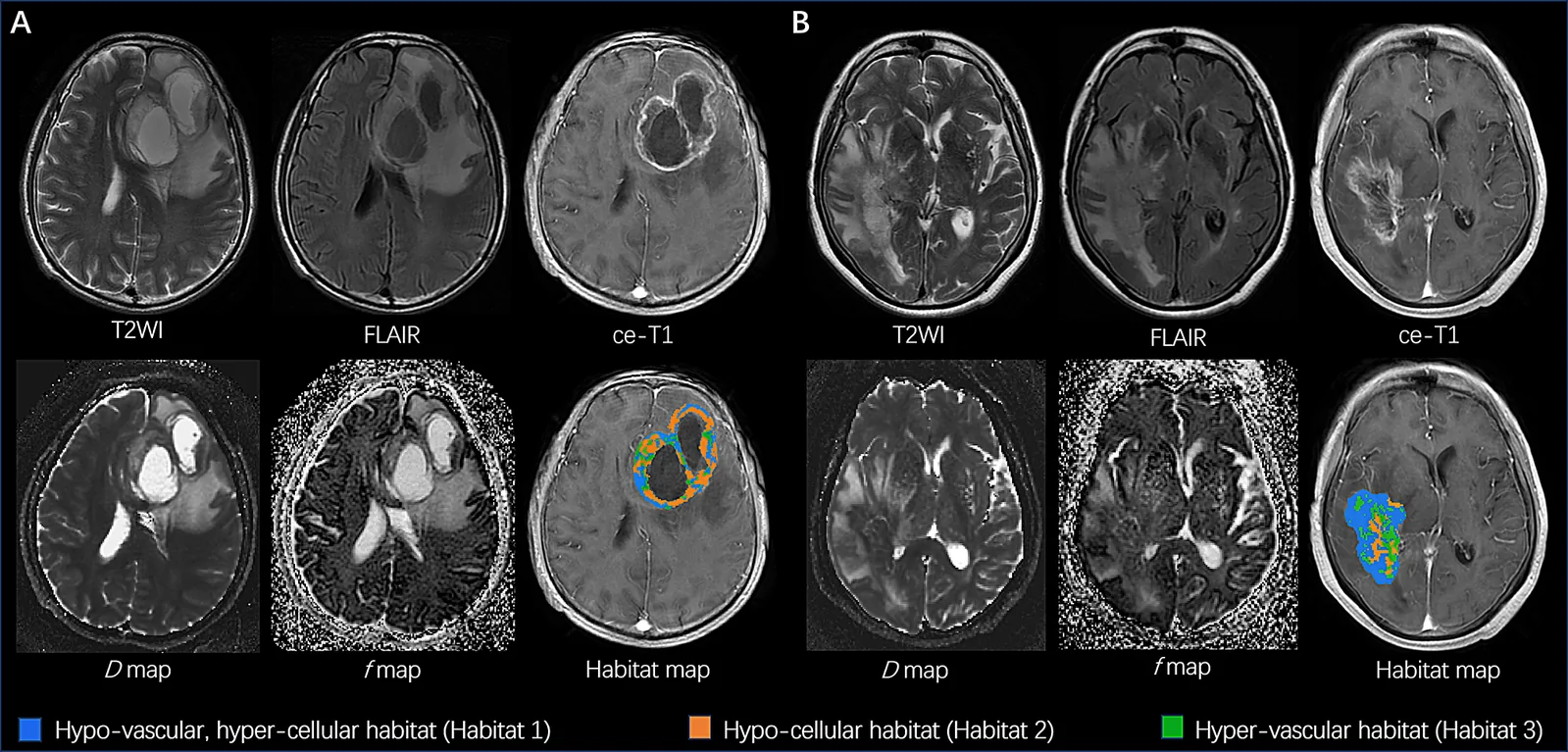
Illustrative examples of spatial habitat slices from two patients with high-grade gliomas. (**A**) A patient with glioblastoma (WHO grade 4) who underwent gross total resection; overall survival 36 months; the hypo-vascular, hyper-cellular habitat occupied 34.4% of tumor volume. (**B**) A second patient with glioblastoma (WHO grade 4) who underwent subtotal resection; overall survival 15 months; the hypo-vascular, hyper-cellular habitat occupied 63.1% of tumor volume. T2WI, T2-weighted imaging; FLAIR, fluid-attenuated inversion recovery; ce-T1, contrast-enhanced T1-weighted imaging; *D*, true diffusion coefficient; *f*, perfusion fraction

After performing k-means clustering, all *D* and *f* voxels were organized into three spatially mapped clusters, representing distinct tumor habitats. Figure 2 illustrates the segmentation results in two typical HGG patients, with the habitats labeled in different colors.

#### *Univariate analysis*

**Fig. 3:**
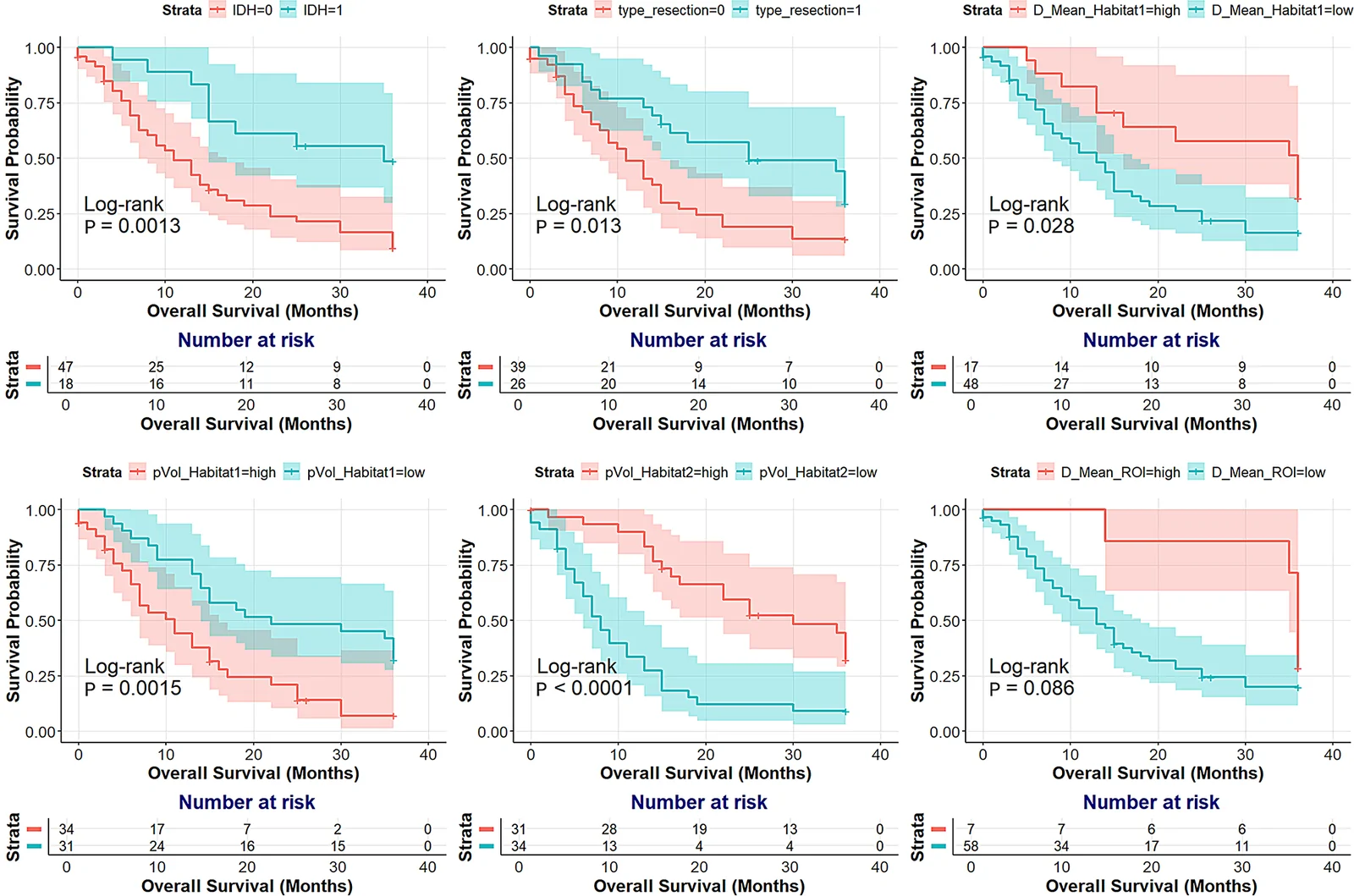
Kaplan-Meier curves for patients stratified by variables identified as significant in univariate Cox analyses. IDH, isocitrate dehydrogenase (1 = mutant, 0 = wild-type); type_resection, resection type (1 = gross total resection, 0 = subtotal resection); pVol, volume percentage; ROI, region of interest

In univariate Cox regression analysis, IDH mutation status, resection type, *D*_mean_ in Habitat 1, pVol of Habitats 1 and 2, and *D*_mean_ in tumor ROI showed associations with OS (Table 3). Thresholds of image features that best split the sample into significant subsets were as follows: 0.892 × 10^−3^ mm^2^/s for *D*_mean_ in Habitat 1 (HR = 0.729, P = 0.022), 47.6% for pVol of Habitat 1 (HR = 5.625, P = 0.006), 15.2% for pVol of Habitat 2 (HR = 0.179, P = 0.014), and 1.225 × 10^−3^ mm^2^/s for *D*_mean_ in tumor ROI (HR = 0.658, P = 0.007). The differences in median OS between the subgroups were 24, 14, 23, 23, 11, and 22 months for IDH, resection type, *D*_mean_ in Habitat 1, *D*_mean_ in tumor ROI, pVol of Habitat 1, and pVol of Habitat 2, respectively. Figure 3 shows the Kaplan–Meier curves for patients stratified according to statistically significant variables.

**Table 3:** Results of univariate cox and kaplan-meier analysis for HGGs

Variable	Optimal threshold	Number of patients below threshold	Number of patients above threshold	Hazard ratio (95% CI) ^#^	*P* Value
Age (y)	67	49	16	1.008 (0.988, 1.028)	0.444
Sex (1/0) ^a^	N/A	24	41	1.023 (0.567, 1.844)	0.940
IDH (1/0) ^b^	N/A	47	18	0.329 (0.159, 0.681)	**0.003**
Resection type (1/0) ^c^	N/A	39	26	0.486 (0.268, 0.882)	**0.018**
Habitat 1 (hypo-vascular, hyper-cellular)					
* D*_mean_ (×10^− 3^mm^2^/s)	0.892	48	17	0.729 (0.555, 0.956)	**0.022**
* f*_mean_ (%)	12.42	40	25	1.008 (0.773, 1.315)	0.953
Volume (cm^3^)	2.83	6	59	1.000 (0.989, 1.011)	0.990
pVol (%)	47.6	31	34	5.625 (1.652, 19.151)	**0.006**
Habitat 2 (hypo-cellular)					
* D*_mean_ (×10^− 3^mm^2^/s)	1.376	31	34	0.871 (0.688, 1.103)	0.252
* f*_mean_ (%)	12.03	36	29	1.008 (0.766, 1.328)	0.953
Volume (cm^3^)	11.88	47	18	0.984 (0.966, 1.002)	0.086
pVol (%)	15.2	34	31	0.179 (0.045, 0.706)	**0.014**
Habitat 3 (hyper-vascular)					
* D*_mean_ (×10^− 3^mm^2^/s)	0.921	57	8	0.892 (0.716, 1.111)	0.307
* f*_mean_ (%)	30.35	58	7	1.002 (0.795, 1.264)	0.986
Volume (cm^3^)	19.90	47	18	0.996 (0.983, 1.010)	0.621
pVol (%)	49.7	45	20	0.956 (0.356, 2.567)	0.928
Tumor ROI					
* D*_mean_ (×10^− 3^mm^2^/s)	1.226	58	7	0.658 (0.485, 0.891)	**0.007**
* f*_mean_ (%)	19.67	45	20	0.989 (0.765, 1.278)	0.932
Volume (cm^3^)	84.03	54	11	0.996 (0.989, 1.003)	0.265

HGG, high-grade glioma; IDH, isocitrate dehydrogenase; *D*, true diffusion coefficient; *f*, perfusion fraction; pVol, volume percentage; ROI, region of interest; CI, confidence interval; N/A, not applicable.^a^1 = male, ^b^1 = IDH mutant status, ^c^1 = gross total resection; Category 0 was used as references.^**#**^ Hazard ratios are for one standard deviation increase in *D*_mean_ and *f*_mean_ measures. Significant *P* values are highlighted in bold

#### Multivariate analysis

**Fig. 4:**
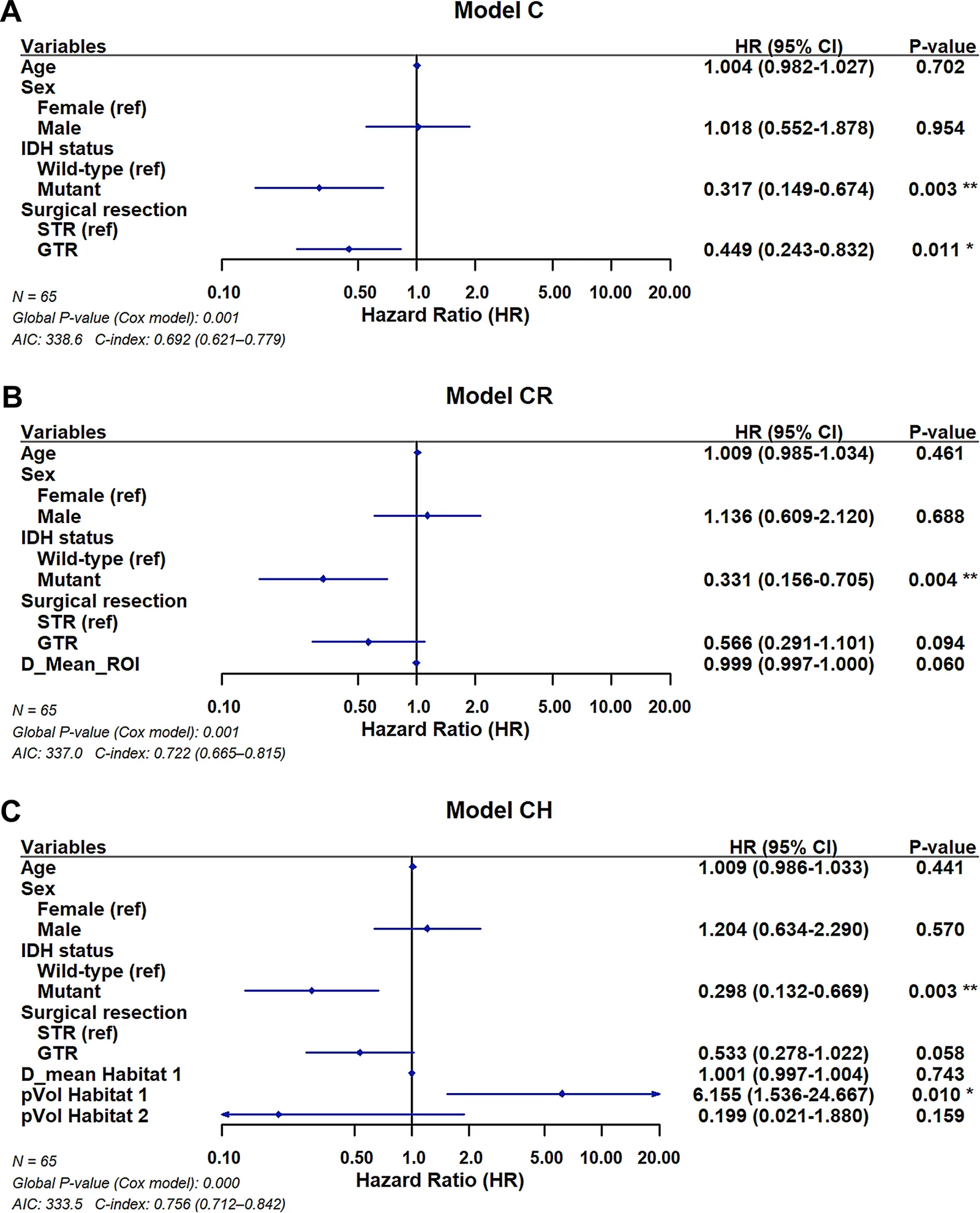
Forest plots of multivariate Cox proportional hazards regression models: (**A**) Model C, based on clinical variables alone; (**B**) Model CR, combining clinical variables with tumor region-of-interest (ROI) features; (**C**) Model CH, incorporating clinical variables and habitat features. IDH, isocitrate dehydrogenase; pVol, volume percentage; HR, hazard ratio; CI, confidence interval; ^**^P < 0.01, ^*^P < 0.05

 Multivariable Cox regression analyses are presented in Fig. 4(A-C). Age, sex, and variables identified in univariate analyses were initially considered for inclusion in the multivariable models. No significant multicollinearity was observed among predictors, with all VIFs < 5. In the clinical-habitat model (Model CH, Fig. 4C), age, sex, IDH genotype, extent of surgical resection, *D*_mean_ in Habitat 1, pVol of Habitat 1, and pVol of Habitat 2 were retained. IDH genotype (HR = 0.298, 95% CI: 0.132–0.669, P = 0.003) and pVol of Habitat 1 (HR = 6.155, 95% CI: 1.536–24.667, P = 0.010) remained independently associated with overall survival. Model CH yielded a C-index of 0.756 (95% CI: 0.712–0.842) and the lowest AIC value (333.5), compared with 0.692 (95% CI: 0.621–0.779; AIC = 338.6) for the clinical model (Model C, Fig. 4A) and 0.722 (95% CI: 0.665–0.815; AIC = 337.0) for the clinical-ROI model (Model CR, Fig. 4B). In paired bootstrap analyses with 1,000 resamples, Model CH demonstrated improved prognostic discrimination relative to Model C (P = 0.006), while showing a nonsignificant trend toward improved performance compared with Model CR (P = 0.056)

### Subgroup analysis

**Fig. 5:**
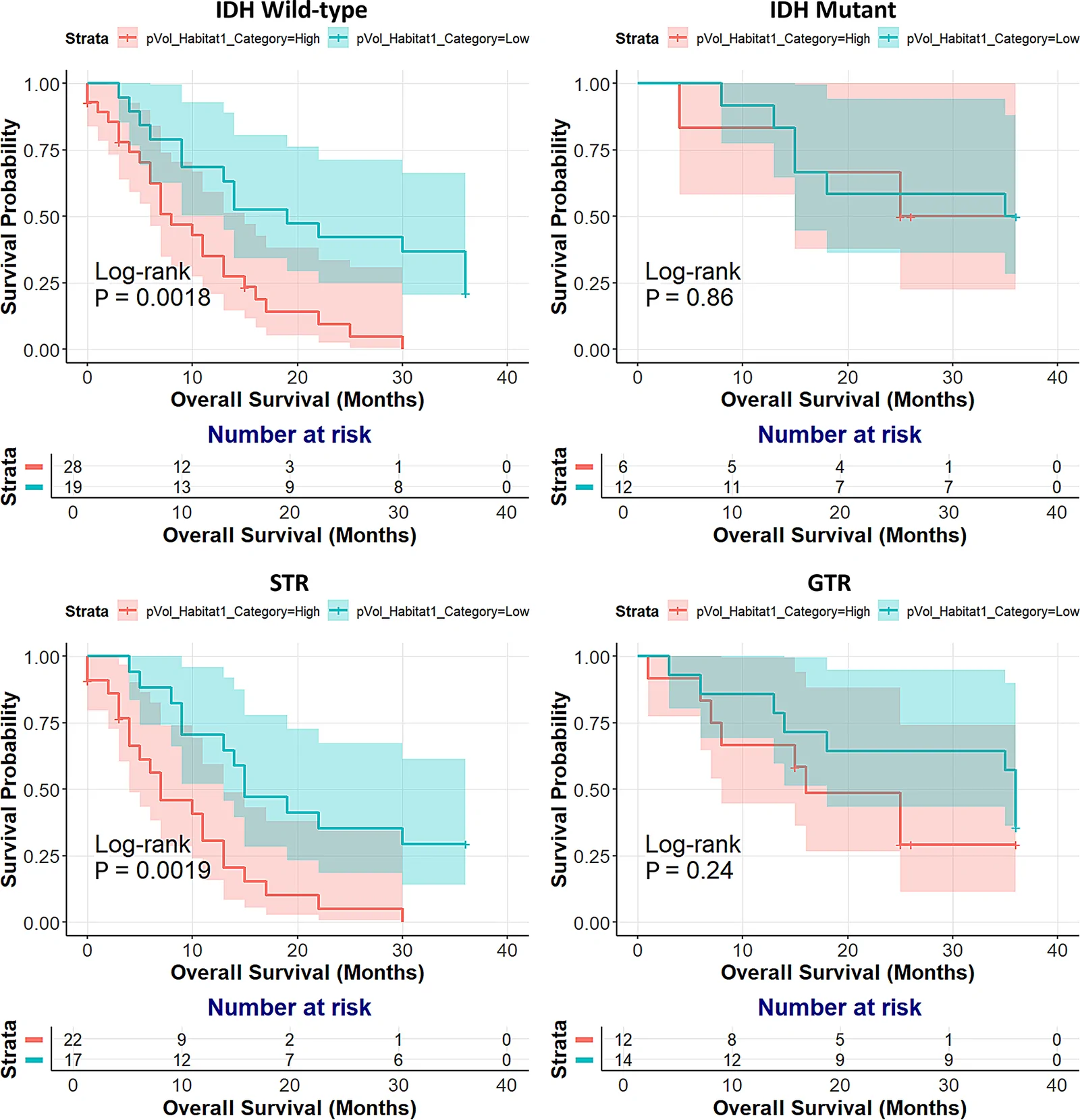
Kaplan-Meier survival curves stratified by volume proportion (pVol) of Habitat 1 in different subgroups. Patients were dichotomized using the previously determined pVol threshold. IDH, isocitrate dehydrogenase; STR, subtotal resection; GTR, gross total resection; pVol, volume percentage

We independently performed Kaplan-Meier analyses in different subgroups based on IDH genotype and resection type. The patients in each subgroup were split by the previously obtained pVol threshold from Habitat 1. In the IDH wild-type and STR subgroups, the differences in survival time between patients with high and low pVol of Habitat 1 were statistically significant. Patients with the high- and low-pVol values in these two subgroups showed median OS differences of 11 and 8 months (HR = 2.950, 95% CI:1.435–6.066, *P* = 0.003; HR = 2.979, 95% CI: 1.421–6.245, *P* = 0.004), respectively. No significant difference was observed in the IDH mutant or GTR subgroups. Figure 5 displays survival curves for the different patient subgroups.

## Discussion

In this study, we evaluated the feasibility of using IVIM MRI-based spatially explicit habitat analysis to characterize ITH in vascularity and cellularity in HGGs and to predict OS. Image voxels representing the solid portion of HGGs were clustered into three distinct physiological subregions: hypo-vascular, hyper-cellular habitat, hypo-cellular habitat, and hyper-vascular habitat. Our findings demonstrated that the volume percentage of the hypo-vascular, hyper-cellular habitat was an independent predictor of patient outcomes. Kaplan-Meier analysis further revealed significant differences in survival between the stratified patient groups. These results suggest that spatially explicit analysis based on IVIM MRI may offer unique and valuable insights into prognostic assessment in HGGs.

Although imaging heterogeneity parameters, such as diffusion and perfusion, have been confirmed to carry prognostic information for HGGs [26], their integration into clinical practice remains challenging. Most studies focus on either diffusion or perfusion alone, often neglecting combined diffusion-perfusion analyses, which may fail to capture the interplay of different biological characteristics and reduce sensitivity to subtle patterns within functional imaging data [27]. Furthermore, current mainstream methods, including histogram-based or radiomics approaches, primarily quantify tumor heterogeneity and complexity by analyzing voxel relationships within a pre-delineated ROI, without considering the spatial distribution of voxels [28]. As a result, these methods may be incapable of fully characterizing ITH, as they typically assume that the tumor is heterogeneous but well mixed [4]. Spatially explicit habitat analysis, which employs clustering algorithms to define spatially distinct subregions with similar physiological characteristics, holds potential to address this limitation [8, 9]. This approach allows for the integration of all extracted imaging values into cluster-based variables, facilitating the detection of naturally occurring hidden patterns within the functional imaging data and the identification of functionally coherent tumor subregions.

We found that the hypo-vascular and hyper-cellular habitat (Habitat 1, defined by low *f* and low *D* values) was associated with OS (pVol: HR = 5.625, *P* = 0.006; *D*_mean_: HR = 0.729, *P* = 0.022) in patients with HGGs. Moreover, multivariable regression analysis adjusted for clinical factors identified pVol as an independent prognostic factor negatively associated with OS (HR = 6.155, *P* = 0.010), indicating that a higher proportion of Habitat 1 predicted worse overall survival. Consistent with our findings, previous studies employing DSC-based habitat imaging have demonstrated a significant association between low relative cerebral blood volume (rCBV) and low ADC habitat (also referred to as the hypo-vascular and hyper-cellular subregion) and patient outcomes in HGGs [29, 30]. For example, Wu et al. [29] reported significant differences in survival between HGG patients stratified by rCBV within low-angiogenic tumor habitats. However, we did not observe a significant association between the perfusion-related parameter *f* and OS, either at the habitat level or across the entire tumor ROI. In fact, the prognostic value of this IVIM-derived metric in HGG remains inconclusive and should be interpreted with caution. While some studies have explored the relationship between perfusion-related metrics and survival, their findings have been inconsistent, with several reporting non-significant associations [31–33], in agreement with our observations. In contrast, diffusion-related parameters reflecting cellular density have demonstrated more consistent associations with tumor aggressiveness and clinical outcomes. For instance, a recent study using voxel-wise clustering of DWI- and DCE-derived metrics showed that lower ADC values in low-vasopermeability, high-cellularity habitats were significantly associated with shorter OS in glioma patients [11]. Several factors may explain this discrepancy. From a methodological perspective, the parameter *f* is more sensitive to acquisition protocols, particularly to the sampling of low b-values, and is more susceptible to noise and fitting instability than parameter *D* [16]. From a biological standpoint, diffusion-related features reflecting cellular density may play a more dominant role in distinguishing tumor subregions in HGG, whereas perfusion heterogeneity may be more spatially diffuse or less distinctly captured under the current imaging conditions. In reality, IVIM reflects the contribution of microvascular flow within each voxel, and its relationship with underlying microvascular histology remains incompletely understood and requires further validation.

The significance of objective tumor volume measurement has been emphasized in evaluating the survival of patients with gliomas [34]. The habitat-driven approach, in which clustering is performed directly on tumor voxels or pixel-level features, allows quantification of distinct components within both non-enhancing and contrast-enhancing tumor regions and facilitates improved characterization of ITH [35]. Kim et al. [24] investigated the temporal dynamics of spatial habitats within glioblastoma and found that an increase in the “hypo-vascular cellular habitat” —characterized by low perfusion and high tumor cell density—was predictive of tumor progression. In our study, we observed that patients with HGGs who had a pVol < 47.6% in the hypo-vascular and hyper-cellular habitat tended to exhibit longer OS, suggesting that spatial heterogeneity captured by IVIM at specific stages of disease progression may serve as a potential tool for prognostic stratification. This habitat likely represents a tumor subpopulation in which cells may adapt to ischemic and hypoxic microenvironments [21, 24]. A previous study reported the presence of angiogenesis-independent tumor growth driven by stem-like cancer cells within heterogeneous tumor subpopulations, contributing to tumor progression [36]. Tumor cells in this region, under ischemic or hypoxic stress, are likely involved in enhanced invasiveness and may promote tumor progression and treatment resistance [24, 37]. Moreover, the hypo-vascular, hyper-cellular habitat may pose challenges for achieving maximal surgical resection, as delineating the precise extent of the peritumoral brain zone (PBZ) preoperatively or intraoperatively is difficult. Tumor cell infiltration into the PBZ has been reported in approximately 35–45% of cases, or even more [38]. Despite significant technical improvements such as micro-neurosurgical resection and contrast agents for enhanced fluorescence-guided surgery, macroscopic GTR is still limited, and more than 80% of cases have recurrences at the edge of the resection cavity several months after treatment [39].

Generally, GTR offers greater benefits than STR [40]. However, conventional imaging approaches have a limited ability to determine the peritumoral infiltration status in HGG patients. In the STR subgroup, the residual hypo-vascular, hyper-cellular tumor tissue may exert a greater influence on survival due to its inherently difficult-to-resect nature. As shown in our subgroup analyses, the pVol of this habitat predicted patient outcomes in the STR subgroup but was not significant in the GTR subgroup, suggesting a therapeutic dilemma regarding the optimal extent of resection and management of tumor infiltration. In brief, future research and treatment strategies should place greater emphasis on the PBZ and on non-enhancing regions surrounding the contrast-enhancing tumor core.

We also observed distinct prognostic implications for Habitat 2 and Habitat 3. Univariate Cox analysis showed that the pVol of Habitat 2, characterized by relatively higher *D* values, exhibited an effect on OS opposite to that of Habitat 1. Physiologically, higher *D* values generally indicate lower cellular density, suggesting that Habitat 2 may be indicative of less proliferative tumor regions or transitional zones within the heterogeneous tumor microenvironment [11]. Consequently, tumors with a higher proportion of Habitat 2 showed more favorable outcomes. In contrast, Habitat 3 comprised voxels with high *f* values but heterogeneous *D* values, which may represent a mixture of perfusion-rich and cellularity-diverse tumor components. Its proportional volume was not significantly associated with OS in our cohort, indicating that although perfusion heterogeneity is captured, it may not independently provide strong prognostic information under the current imaging conditions. Collectively, these observations underscore the dominant role of diffusion-derived features in defining prognostically relevant tumor habitats, whereas perfusion-related metrics, such as *f*, appear complementary but less discriminative. Interpretations should remain cautious, as both diffusion and perfusion parameters provide indirect measures of tumor biology, warranting further validation with histopathology and molecular characterization [15, 16].

This study has several limitations. First, the relatively small single-center cohort and the exploratory nature of the analyses may limit the statistical robustness and generalizability of the findings. The prognostic models were developed and evaluated within the same cohort without external validation, train/test splitting, or cross-validation, and the use of data-driven optimal cutoff methods may have introduced overfitting and optimistic bias. Second, WHO grade 3 and grade 4 gliomas were analyzed together despite their known biological and prognostic heterogeneity, while the limited number of grade 3 tumors precluded reliable subgroup analyses and longer-term survival assessment. Third, direct spatial histopathological validation of the imaging-derived habitats was not feasible because of the difficulty in obtaining spatially matched tissue samples. Therefore, the present findings should be considered preliminary exploratory evidence from a feasibility study and require further validation in larger prospective multicenter cohorts.

In conclusion, spatially explicit habitat analysis based on IVIM MRI enables preoperative characterization of perfusion- and diffusion-related heterogeneity in HGGs. Among the identified habitats, the hypo-vascular, hyper-cellular compartment was associated with overall survival, suggesting potential prognostic relevance. These findings highlight the utility of spatially explicit IVIM MRI for noninvasive assessment of tumor heterogeneity and provide a foundation for future studies exploring its role in risk stratification and personalized therapeutic strategies.

## Supplementary Information

Below is the link to the electronic supplementary material.

Supplementary Material 1

## Data Availability

Data are provided within the manuscript or supplementary information file. Detailed datasets used and/or analyzed during the current study are available from the corresponding author upon reasonable request.

## Abbreviations

IVIM: Intravoxel incoherent motion
MRI: Magnetic resonance imaging
HGG: High-grade glioma
ITH: Intratumor heterogeneity
OS: Overall survival
ROI: Region of interest
*D*: True diffusion coefficient
*f*: Perfusion fraction
pVol: Volume percentage
IDH: Isocitrate dehydrogenase
GTR: Gross total resection
STR: Subtotal resection

## References

[1] Weller M, Wen PY, Chang SM, Dirven L, Lim M, Monje M, Reifenberger G. Glioma. Nat Reviews Disease Primers. 2024;10(1):33. 38724526 10.1038/s41572-024-00516-y

[2] Nicholson JG, Fine HA. Diffuse Glioma Heterogeneity and Its Therapeutic Implications. Cancer Discov. 2021;11(3):575–90. 33558264 10.1158/2159-8290.CD-20-1474

[3] Lauko A, Lo A, Ahluwalia MS, Lathia JD. Cancer cell heterogeneity & plasticity in glioblastoma and brain tumors. Semin Cancer Biol. 2022;82:162–75. 33640445 10.1016/j.semcancer.2021.02.014 PMC9618157

[4] Gatenby RA, Grove O, Gillies RJ. Quantitative Imaging in Cancer Evolution and Ecology. Radiology. 2013;269(1):8–15. 24062559 10.1148/radiol.13122697 PMC3781355

[5] Hambardzumyan D, Bergers G. Glioblastoma: Defining Tumor Niches. Trends Cancer. 2015;1(4):252–65. 27088132 10.1016/j.trecan.2015.10.009 PMC4831073

[6] Esmaeili M, Vettukattil R, Bathen TF. 2-hydroxyglutarate as a magnetic resonance biomarker for glioma subtyping. Transl Oncol. 2013;6(2):92–8. 23544162 10.1593/tlo.12424 PMC3610550

[7] van den Bent MJ, Geurts M, French PJ, Smits M, Capper D, Bromberg JEC, Chang SM. Primary brain tumours in adults. Lancet. 2023;402(10412):1564–79. 37738997 10.1016/S0140-6736(23)01054-1

[8] Napel S, Mu W, Jardim-Perassi BV, Aerts HJWL, Gillies RJ. Quantitative imaging of cancer in the postgenomic era: Radio(geno)mics, deep learning, and habitats. Cancer. 2018;124(24):4633–49. 30383900 10.1002/cncr.31630 PMC6482447

[9] O’Connor JPB, Rose CJ, Waterton JC, Carano RAD, Parker GJM, Jackson A. Imaging Intratumor Heterogeneity: Role in Therapy Response, Resistance, and Clinical Outcome. Clin Cancer Res. 2015;21(2):249–57. 25421725 10.1158/1078-0432.CCR-14-0990 PMC4688961

[10] Moon HH, Park JE, Kim N, Park SY, Kim YH, Song SW, Hong CK, Kim JH, Kim HS. Prospective longitudinal analysis of physiologic MRI-based tumor habitat predicts short-term patient outcomes in IDH-wildtype glioblastoma. Neuro Oncol. 2025;27(3):841–53. 39450860 10.1093/neuonc/noae227 PMC11889713

[11] Wang X, Wu H, Wang Y, Hu W, Suo S, Guo L, et al. Preoperative prediction of Ki-67 expression and risk stratification in gliomas using multiparametric MRI and intratumor heterogeneity-based habitat imaging: a multicenter study. Int J Surg. 2026;112(2):2554–68. 10.1097/JS9.0000000000003766 41133387

[12] Herings S, de Wit R, Saglik B, Mannil M, van den Elshout R, Arens A, et al. Quantification of fractional tumor burden for the early detection of post-treatment glioblastoma progression. Eur Radiol Exp. 2026;10(1):21. 10.1186/s41747-026-00685-3 PMC12953891 41770432

[13] Li AY, Iv M. Conventional and Advanced Imaging Techniques in Post-treatment Glioma Imaging. Front Radiol. 2022;2:883293. 37492665 10.3389/fradi.2022.883293 PMC10365131

[14] Hirschler L, Sollmann N, Schmitz-Abecassis B, Pinto J, Arzanforoosh F, Barkhof F, Booth T, Calvo-Imirizaldu M, Cassia G, Chmelik M, et al. Advanced MR Techniques for Preoperative Glioma Characterization: Part 1. J Magn Reson Imaging. 2023;57(6):1655–75. 36866773 10.1002/jmri.28662 PMC10946498

[15] Iima M. Perfusion-driven Intravoxel Incoherent Motion (IVIM) MRI in Oncology: Applications, Challenges, and Future Trends. Magn Reson Med Sci. 2021;20(2):125–38. 32536681 10.2463/mrms.rev.2019-0124 PMC8203481

[16] Sigmund EE, Rauh SS, Iima M, Federau C, Hernando D, Jalnefjord O, et al. Towards clinical translation of intravoxel incoherent motion MRI: acquisition and analysis consensus recommendations. J Magn Reson Imaging. 2026;63(6):1782–1801. 10.1002/jmri.70278 PMC13175230 41853873

[17] Le Bihan D, Breton E, Lallemand D, Aubin ML, Vignaud J, Laval-Jeantet M. Separation of diffusion and perfusion in intravoxel incoherent motion MR imaging. Radiology. 1988;168(2):497–505. 3393671 10.1148/radiology.168.2.3393671

[18] Le Bihan D, Turner R. The capillary network: a link between IVIM and classical perfusion. Magn Reson Med. 1992;27(1):171–8. 1435202 10.1002/mrm.1910270116

[19] Zhou M, Hall L, Goldgof D, Russo R, Balagurunathan Y, Gillies R, Gatenby R. Radiologically defined ecological dynamics and clinical outcomes in glioblastoma multiforme: preliminary results. Transl Oncol. 2014;7(1):5–13. 24772202 10.1593/tlo.13730 PMC3998688

[20] Sheng Y, Dang X, Zhang H, Rui W, Wang J, Cheng H, Qiu T, Zhang Y, Ding Y, Yao Z, et al. Correlations between intravoxel incoherent motion-derived fast diffusion and perfusion fraction parameters and VEGF- and MIB-1-positive rates in brain gliomas: an intraoperative MR-navigated, biopsy-based histopathologic study. Eur Radiol. 2023;33(8):5236–46. 36941492 10.1007/s00330-023-09506-2

[21] Wang XR, Xie ZH, Wang XQ, Song Y, Suo ST, Ren Y, Hu WT, Zhu Y, Cao MQ, Zhou Y. Preoperative prediction of IDH genotypes and prognosis in adult-type diffuse gliomas: intratumor heterogeneity habitat analysis using dynamic contrast-enhanced MRI and diffusion-weighted imaging. Cancer Imaging. 2025;25(1):11. 39923105 10.1186/s40644-025-00829-5 PMC11807326

[22] Le Bihan D, Breton E, Lallemand D, Grenier P, Cabanis E, Laval-Jeantet M. MR imaging of intravoxel incoherent motions: application to diffusion and perfusion in neurologic disorders. Radiology. 1986;161(2):401–7. 3763909 10.1148/radiology.161.2.3763909

[23] Song Y, Zhang J, Zhang Y-d, Hou Y, Yan X, Wang Y, Zhou M, Yao Y-f, Yang G. FeAture Explorer (FAE): A tool for developing and comparing radiomics models. PLoS ONE. 2020;15(8):e0237587. 32804986 10.1371/journal.pone.0237587 PMC7431107

[24] Kim M, Park JE, Kim HS, Kim N, Park SY, Kim YH, Kim JH. Spatiotemporal habitats from multiparametric physiologic MRI distinguish tumor progression from treatment-related change in post-treatment glioblastoma. Eur Radiol. 2021;31(8):6374–83. 33569615 10.1007/s00330-021-07718-y

[25] Pérez-Beteta J, Molina-García D, Ortiz-Alhambra JA, Fernández-Romero A, Luque B, Arregui E, et al. Rodríguez de Lope Á. Tumor surface regularity at MR imaging predicts survival and response to surgery in patients with glioblastoma. Radiology. 2018;288(1):218-225. 10.1148/radiol.2018171051 29924716

[26] Hu LS, Hawkins-Daarud A, Wang L, Li J, Swanson KR. Imaging of intratumoral heterogeneity in high-grade glioma. Cancer Lett. 2020;477:97–106. 32112907 10.1016/j.canlet.2020.02.025 PMC7108976

[27] Foltyn-Dumitru M, Kessler T, Sahm F, Wick W, Heiland S, Bendszus M, Vollmuth P, Schell M. Cluster-based prognostication in glioblastoma: Unveiling heterogeneity based on diffusion and perfusion similarities. Neuro Oncol. 2024;26(6):1099–108. 38153923 10.1093/neuonc/noad259 PMC11145444

[28] Park JE, Kim HS, Kim N, Park SY, Kim YH, Kim JH. Spatiotemporal Heterogeneity in Multiparametric Physiologic MRI Is Associated with Patient Outcomes in IDH-Wildtype Glioblastoma. Clin Cancer Res. 2021;27(1):237–45. 33028594 10.1158/1078-0432.CCR-20-2156

[29] Wu H, Tong H, Du X, Guo H, Ma Q, Zhang Y, Zhou X, Liu H, Wang S, Fang J, et al. Vascular habitat analysis based on dynamic susceptibility contrast perfusion MRI predicts IDH mutation status and prognosis in high-grade gliomas. Eur Radiol. 2020;30(6):3254–65. 32078014 10.1007/s00330-020-06702-2

[30] Liu J, Cong C, Zhang J, Qiao J, Guo H, Wu H, Sang Z, Kang H, Fang J, Zhang W. Multimodel habitats constructed by perfusion and/or diffusion MRI predict isocitrate dehydrogenase mutation status and prognosis in high-grade gliomas. Clin Radiol. 2024;79(1):e127–36. 37923627 10.1016/j.crad.2023.09.025

[31] Qiu J, Zhu M, Chen CY, Luo Y, Wen J. Diffusion heterogeneity and vascular perfusion in tumor and peritumoral areas for prediction of overall survival in patients with high-grade glioma. Magn Reson Imaging. 2023;104:23–8. 37734575 10.1016/j.mri.2023.09.004

[32] Jabehdar Maralani P, Myrehaug S, Mehrabian H, Chan AKM, Wintermark M, Heyn C, Conklin J, Ellingson BM, Rahimi S, Lau AZ, et al. Intravoxel incoherent motion (IVIM) modeling of diffusion MRI during chemoradiation predicts therapeutic response in IDH wildtype glioblastoma. Radiother Oncol. 2021;156:258–65. 33418005 10.1016/j.radonc.2020.12.037 PMC8186561

[33] Federau C, Cerny M, Roux M, Mosimann PJ, Maeder P, Meuli R, Wintermark M. IVIM perfusion fraction is prognostic for survival in brain glioma. Clin Neuroradiol. 2017;27(4):485–92. 27116215 10.1007/s00062-016-0510-7

[34] Kickingereder P, Isensee F, Tursunova I, Petersen J, Neuberger U, Bonekamp D, Brugnara G, Schell M, Kessler T, Foltyn M, et al. Automated quantitative tumour response assessment of MRI in neuro-oncology with artificial neural networks: a multicentre, retrospective study. Lancet Oncol. 2019;20(5):728–40. 30952559 10.1016/S1470-2045(19)30098-1

[35] Wu J, Xia Y, Wang X, Shi F, Shen D. Radiomics++: review of habitat imaging analysis for decoding tumor heterogeneity. Annu Rev Biomed Eng. 2026;28(1):219–48. 10.1146/annurev-bioeng-031825-040442 41591802

[36] Sakariassen PØ, Prestegarden L, Wang J, Skaftnesmo K-O, Mahesparan R, Molthoff C, et al. Angiogenesis-independent tumor growth mediated by stem-like cancer cells. Proc Natl Acad Sci U S A. 2006;103(44):16466-16471. 10.1073/pnas.0607668103 PMC1618812 17056721

[37] Zhang G, Tao X, Ji BW, Gong J. Hypoxia-Driven M2-Polarized Macrophages Facilitate Cancer Aggressiveness and Temozolomide Resistance in Glioblastoma. Oxidative Med Cell Longev. 2022;2022:1–20. 10.1155/2022/1614336 PMC9423979 36046687

[38] Trevisi G, Mangiola A. Current Knowledge about the Peritumoral Microenvironment in Glioblastoma. Cancers. 2023;15(22):5460. 38001721 10.3390/cancers15225460 PMC10670229

[39] Ballestín A, Armocida D, Ribecco V, Seano G. Peritumoral brain zone in glioblastoma: biological, clinical and mechanical features. Front Immunol. 2024;15:1347877. 38487525 10.3389/fimmu.2024.1347877 PMC10937439

[40] Brown TJ, Brennan MC, Li M, Church EW, Brandmeir NJ, Rakszawski KL, Patel AS, Rizk EB, Suki D, Sawaya R, et al. Association of the Extent of Resection With Survival in Glioblastoma A Systematic Review and Meta-analysis. Jama Oncol. 2016;2(11):1460–9. 27310651 10.1001/jamaoncol.2016.1373 PMC6438173

