# Supplementary material for: Preoperative assessment of intratumor heterogeneity using intravoxel incoherent motion MRI for survival prediction in high-grade gliomas: a feasibility study
Source: BMC Med Imaging. 2026 Jun 12;26:421. doi: 10.1186/s12880-026-02499-6 (PMC13508330; doi:10.1186/s12880-026-02499-6)
Supplement: Supplementary file 1 [file 12880_2026_2499_MOESM1_ESM.docx]

**Preoperative Assessment of Intratumor Heterogeneity Using Intravoxel Incoherent Motion MRI for Survival Prediction in High-Grade Gliomas: A Feasibility Study**

**Supplementary Material**

**Supplemental Fig. 1** Evaluation of the optimal number of clusters (k) for habitat analysis. To determine the optimal number of clusters for k-means-based habitat segmentation, we systematically evaluated solutions with k ranging from 2 to 5 (**A**). For each k, we calculated the sum of squared errors (SSE), which quantifies the total within-cluster variance, and the silhouette score, which measures the consistency of clustering by assessing how similar a voxel is to its own cluster compared to other clusters (**B**). As expected, SSE decreased as k increased, reflecting improved within-cluster homogeneity. However, the rate of decrease diminished after k = 3, indicating diminishing returns from increasing cluster number. The silhouette score reached its maximum (0.361) at k = 3, suggesting the best balance between cluster cohesion and separation. Taken together, these results indicate that k = 3 provides an optimal trade-off between minimizing within-cluster variance, maintaining cluster distinctness, and preserving biological interpretability.

**Supplementary Table S1** Individual Dice similarity coefficients for inter-observer tumor segmentation

| 0.829 | 0.881 | 0.831 | 0.873 | 0.791 | 0.894 | 0.831 | 0.857 | 0.824 | 0.869 |
| --- | --- | --- | --- | --- | --- | --- | --- | --- | --- |
| 0.838 | 0.856 | 0.812 | 0.870 | 0.869 | 0.885 | 0.801 | 0.810 | 0.826 | 0.877 |
